# The Effect of Emotional and Cultural Intelligences on Networks’ Behaviors in International SMEs: Evidence from Portugal

**DOI:** 10.3390/bs10110163

**Published:** 2020-10-24

**Authors:** Ângelo Miguel R. Cabral, Fernando Manuel P. O. Carvalho, José António V. Ferreira

**Affiliations:** 1 CeBER, Faculty of Economics, University of Coimbra, 3004-512 Coimbra, Portugal; fc@fe.uc.pt; 2 GOVCOPP, Department of Economics, Management, Industrial Engineering and Tourism, University of Aveiro, 3810-193 Aveiro, Portugal; josev@ua.pt

**Keywords:** networking, external networking behavior, emotional intelligence, cultural intelligence, small and medium-sized enterprises

## Abstract

The major purpose of this research was to study the predictive value of the top managers’ psychological characteristics regarding their networking behavior. In the international business management context of small- and medium-sized enterprises, we took the top managers’ cultural intelligence and emotional intelligence as determinant capabilities to perform better in their external networking. The sample was composed of 307 Portuguese SMEs’ international decision-makers, specifically founders, owners, chief executive officers (CEOs), managers of international activities, international market managers, or commercial managers. The data was collected from 2–30 April 2019 through online surveys directed to the Portuguese decision-makers that were directly responsible for the firms’ international activities. As a data collection instrument, the surveys were pretested and sent by e-mail. The average age of the participants was approximately 50 years old for males and 45 years old for females. We used self-reported measures to assess the different constructs and the hierarchical regression analysis to test our hypotheses. The results showed that cultural intelligence and emotional intelligence were significant drivers of decision-makers’ external networking behavior. A new factor structure concerning external networking behavior was retained. The major results exhibited the predictive value of some cultural and emotional intelligence dimensions over the new retained external networking behavior factors. Therefore, in the international business management context, the capability to adapt to new cultural contexts, as well as the capability to reason about emotions, improved the international decision-makers’ external networking behavior.

## 1. Introduction

The present research was grounded in the international business management, networks, and psychology fields. In the international management context, this study focused on the networking behavior and psychological characteristics of the international decision-makers of the international small- and medium-sized enterprises (SMEs). The conceptual and empirical approach took decision-makers’ psychological characteristics as important antecedents of their networking behavior in the setting of SMEs’ internationalization.

This research connected the international top managers’ cultural and emotional intelligences to their external networking behaviors, suggesting that the capability to adapt to new cultural contexts, as well as the capability to reason about emotions and to use emotional knowledge to enhance thought, improved their networking behaviors, specifically, in building and maintaining external contacts, in using external contacts in a win–win perspective, and in their commitment to the creation of external contacts. To our knowledge, the investigation concerning the effects of emotional intelligence (EI) and cultural intelligence (CQ) on external networking behaviors is under-researched. According to our research, little is known about the relationship between these two intelligences and external networking behavior. We, therefore, proposed a model, where the main research question was whether the levels of CQ and EI of the international SMEs’ decision-makers were associated with their engagement intensity in their external networking behavior (ENB). This embodies an important line of research due to the importance of networks in the international business management context and due to the relevance of the individuals’ emotional and cultural intelligences to their behaviors.

The present research was grounded in the network approach of SMEs’ internationalization, where Chetty and Holm [1] argued that a firm’s environment is embedded with networking relationships and that those relationships with partners stimulate the necessary dynamics for internationalization. The networking relationships are a valuable asset to the SMEs’ internationalization [2]. Within the networking activity, we also state the individual differences as important antecedents [3]. Furthermore, we relied on emotional intelligence theory, which states this ability is the one that supports accurate reasoning about emotions and the use of emotions to improve thought [4]. We also relied on cultural intelligence theory, which states that cultural intelligence is a “person’s capability to adapt effectively to new cultural contexts” [5] (p. 59). Hence, through these last two theories, the value of the intelligences is applied to boost the international top managers’ external networking behavior.

Internationalization is an important decision taken by SMEs to promote their growth and competitiveness [6]. SMEs are increasingly present in the international environment [7] and undoubtedly an important player in the world economy [8]. However, within this environment, some constraints and challenges emerge, for instance, sociocultural differences, limitations on resources, and competitive challenges [6]. In this context, personal factors appear to have an important role in SMEs’ internationalization [9,10]. Networks and networking activity are crucial to SMEs’ internationalization development and success, allowing them to solve some constraints about resources, to recognize opportunities, to promote access to information, and to help improve competitive advantages [11,12,13,14]. Along this line, relationships via networks are an important ingredient for internationalization [15,16], to which managers must pay special attention to whether in its construction or in its maintenance [17]. It should be noted that networking integrates internal and external behaviors into organizations [3]. This research considered the decision-makers’ external facet of networking behavior and recognized that the networking activity is influenced by individual differences [18]. Regarding the top managers’ psychological characteristics, we took cultural intelligence and emotional intelligence as important capabilities for dealing with international environments. They are two important intelligences within cross-cultural contexts [19]. When firms are international, their international top managers face several emotional and cultural vicissitudes with which they have to deal. Therefore, emotional and cultural intelligences appear to have a decisive role when the international decision-makers are carrying out their responsibilities.

Top managers’ CQ is an important characteristic for those who interact in multicultural settings. Those with higher levels of this intelligence are better prepared for interactions in intercultural environments [20] and for cross-cultural adjustment [21]. CQ has a relevant role in relationships with foreign stakeholders [22] and allows culturally intelligent individuals to be more cooperative in negotiation contexts [23]. Top managers’ EI has an important role within networks and external networking behavior [24]. Our study suggested that those who better reason about emotions and use emotional knowledge to enhance thought [4] are better prepared to boost their networking behaviors. These behaviors are embedded with interpersonal relationships [3], which is a fact that displays EI as an important ability in this domain given its benefits to social interactions [25]. Therefore, in this context, this research suggests that decision-makers’ CQ and EI are significant capabilities in the top managers’ networking behavior outside the firm.

In the context of international business management, like other investigations, our research reinforced the importance and the central role of the top managers’ psychological characteristics [26,27], as well as its importance in their networking behavior [22,28,29]. Networks are an important phenomenon in SMEs’ internationalization, where top managers are faced with a lot of network relationships in the performance of their international roles. Therefore, networking is seen as a relevant behavior with important implications for firms and for the performance of international top managers. In this vein, understanding which drivers of networking behavior are significant and affect the relationships’ performance is an important line of research. The psychological characteristics are an important area of study in the domain where the present research was focused [28]. In fact, at the individual level, the psychological domain was emphasized as it encompasses important drivers for networking [3]. For instance, personality is a significant antecedent that is stressed in the literature [3,28]. In this context, Johanson and Vahlne [30] stressed that the business environment is a network where relationships embody cognitive and emotional dimensions.

As emphasized above, we took CQ and EI as drivers of the international decision-makers’ external networking behavior. CQ contributes to SMEs’ international network ties, helping to build quality relationships with foreign customers, suppliers, and competitors [22]. Its role in the intercultural contexts is identifiable by the fact that culturally intelligent individuals are more prone and prepared to interact with others because they are more capable of cross-cultural adjustment and exhibiting cooperative motives and motivation [21,31,32]. According to Leonidou et al. [29], EI is an important capability that enhances an effective interaction with others, positively impacting the communication, the social bonds, decreasing relational distance, and levels of conflict within the exporter–importer relationships. However, this emotional capability is characterized by a scarcity of studies in cross-border relationships [29].

Concerning the structure of this research, Section 2 is composed of the theoretical foundation regarding external networking behavior, emotional intelligence, cultural intelligence, and the development of the hypotheses. In Section 3, the methodology followed is addressed. In Section 4, we present the empirical results of the hypothesis testing. Section 5 contains the discussion and the theoretical and empirical implications of the research. Finally, Section 6 presents the conclusion and addresses the limitations of this research and proposes future research avenues.

## 2. Theoretical Background and Hypotheses Formulation

### 2.1. External Networking Behavior

The relevance of personal relationships is present in business internationalization, a fact that gives emphasis to the networks as an important subject of research in this context [13,14,30,33]. Top managers’ networking relationships matter and embody a crucial social activity in business reality [16,24]. Networking is seen as a behavioral activity that is important to career success [18], to job performance, and for access to strategic information [3], as well as in overcoming some entrepreneurial constraints [34]. Concerning SMEs, networking is an important internationalization booster and a relevant source of information [2]. It is considered an individual behavior construct where “individuals differ in the amount of networking they engage in” [18] (p. 113). In this research, we focused on the decision-makers’ networking and followed the networking definition of Gibson et al. [3]: “networking is a form of goal-directed behavior, both inside and outside of an organization, focused on creating, cultivating, and utilizing interpersonal relationships” (p. 150). This investment in social relationships [18], besides being an important source of information [35], allows for the recognition of opportunities and problem solving [3,34].

ENB concerns individuals’ behavior with contacts from other organizations, i.e., behaviors about developing relationships outside the firm [35]. ENB is grounded on the approach of networking that considers both an internal and an external facet of it, and within each facet, the process of relationship development through building, maintaining, and using contacts [28]. In fact, these stages of development must be distinguished given that networking is a continuous activity and not a stagnant one [3]. In this study, the decision-makers’ ENB embraced three types of behaviors: building external contacts, maintaining external contacts, and using external contacts [28]. In relation to the antecedents of networking behaviors, individual differences are emphasized in the literature, where the psychological ones are accentuated [3,28].

In the international business management context, to pursue foreign activities and their inherent challenges, managers need some important skills [29]. This research focused precisely on the decision-makers’ psychological characteristics as determinants of their ENB. Leonidou et al. [29] highlight that EI and CQ, and other types of intelligence, may influence a diverse number of international activities. Therefore, given the international context at stake, several multicultural interactions emerge, displaying the importance of CQ. Hence, we took CQ as the capability that enables decision-makers to be more prone and prepared to interact within intercultural contexts [31]. Given that social interactions are the core of networking behaviors [3], EI is a capability that promotes better social outcomes [25] and enhances an effective interaction with others [29], improving its quality [36]. Thus, this study postulated these characteristics as being relevant antecedents of ENB that contribute to a more dynamic network activity of the decision-makers, i.e., those with higher levels in these intelligences engage in networking behaviors more than others.

### 2.2. Emotional Intelligence

In the present research, we focused on the EI of the international SMEs’ decision-makers. It is important to study EI in the organizational context [29,37]. However, in the international business management context, there is a substantial lack of research about its role [21]. 

Within organizations, given that interactions between individuals are a reality, emotions thrive and emotionally intelligent individuals will be better prepared to perform their responsibilities [38]. “Emotional intelligence concerns the ability to carry out accurate reasoning about emotions and the ability to use emotions and emotional knowledge to enhance thought” [4] (p. 511). Following Wong and Law [36], we took EI as a multidimensional construct that is constituted by four dimensions: self-emotions appraisal (SEA), others-emotions appraisal (OEA), use of emotion (UOE), and regulation of emotion (ROE). 

This is an important ability that allows for an effective adjustment of those who operate in multicultural settings [21]. It improves social functioning, and higher-level EI individuals are more socially competent, have better quality in their relationships, are more interpersonally sensitive, and work and communicate better with others [39]. Therefore, we took EI as a significant antecedent of decision-makers’ ENB, given that this behavior involves the development of relationships with individuals outside the organization [35]. In this sense, EI appears to be a major psychological characteristic when managers interact with others to build, maintain, and use their external relationships. Higher-level EI individuals tend to be perceived more positively, e.g., pleasant and empathic [4].

Meeting people outside the organization takes more effort than internal networking [28]. The establishment and creation of new contacts is a challenging and consuming social activity [28]. Maintaining and using contacts is related to interactions with known people, friends, or colleagues [40]. Thus, to managers, these circumstances suggest a determinant role of knowing how to deal with emotions. Emotions influence social interactions, and emotional intelligence, besides promoting a better dynamic with social environments [41], is a determinant capability in the management of relationships [29]. Higher levels of EI help to avoid interpersonal conflicts in social and business contexts [25]. In the international business management context, Leonidou et al. [29] stressed that the exporters’ EI promotes a positive atmosphere in the relationship with import buyers, which enhances communication and social bonding and reduces the relational distance and conflict. According to the results of Delpechitre et al. [42], the higher the abilities of salespeople to use and perceive emotions, the higher the tendency of customers to exhibit customer value co-creation behaviors. In their study, EI also showed an influence over the customers’ commitment to salespeople. Hence, EI has a widespread positive effect in organizational contexts, e.g., job performance [38], individual ethics, ethics and success perceptions [43], entrepreneurial style, external networking behavior [24], and creating win–win situations with others [44]. In diverse life outcomes, the predictive value of EI is identified, among others, in better social relations, better work relationships and negotiations, and better psychological well-being [4]. According to Torres-Coronas and Vidal-Blasco [45], the ability to regulate emotions influences entrepreneurs’ networking behavior in a way that promotes better business relationships. 

Consequently, taking the above into consideration, the present research took EI as a predictor of ENB and its dimensions. Those that possess higher levels in this intelligence perform better than others in terms of external networking behavior. In summary, EI should help to capitalize on decision-makers’ actions toward ENB. Therefore, we formulate Hypothesis 1 (H1) as the following:

**Hypothesis 1** **(H1).** *The higher the level of a decision-maker’s EI, the better their performance regarding their ENB.*

### 2.3. Cultural Intelligence

For those who interact in the international business management context, CQ is a significant capability [46]. It is a critical facilitator for individuals to interact well in cross-cultural business environments [32]. Cross-cultural capabilities are a determinant in international negotiations, which allows for understanding, communication, and behavioral flexibility with different cultures [23]. The cultural diversity present in life in general and in the working context calls for people’s abilities to interact with individuals from different cultures and to interact with the most diverse cultural aspects [19,46,47]. In the international SME context, the interaction with different foreign stakeholders is a reality with which managers have to deal and preferentially with high-quality relationships [22]. According to Earley and Ang [5], “cultural intelligence refers to a person’s capability to adapt effectively to new cultural contexts” (p. 59). Following Ang et al. [48], we took CQ as a multidimensional construct with four dimensions: metacognitive (MC), cognitive (COG), motivational (MOT), and behavioral (BEH).

In relation to networking, its core characteristic is concerned with behaviors toward interpersonal relationships [3] by building and cultivating them [40]. Hence, given the international business management context of the present research, it should be emphasized that “CQ is a critical competency that allows SME entrepreneurs to develop good relationships with foreign network ties” [22] (p. 424). In line with this, some effects should be emphasized given their importance to cross-cultural interactions. According to Imai and Gelfand [32], CQ enhances the effectiveness of intercultural negotiation processes and allows for culturally intelligent individuals to be more confident, motivated, and cooperative in intercultural relationships. CQ also exhibits a positive effect on cross-cultural adjustment [21], which leads to a reduction of cultural shock when contact with different cultures takes place [31]. Higher-level CQ individuals have a better knowledge of different cultural norms, practices, and conventions; are more capable to adapt themselves to intercultural settings; are more easily accepted by their counterparts; are more capable of developing better interpersonal relationships [21]. Therefore, CQ constitutes an important capability regarding managers’ engaging effectively in multicultural interactions, managing conflicts [20], and exhibiting cooperative behaviors in negotiation contexts [23]. According to Charoensukmongkol [22], CQ has an important and positive effect on international network ties, i.e., on the quality of the managers’ relationships with foreign stakeholders. CQ reveals the potential to help communicate better with others in multicultural settings and to build networks with better relationships [22]. Higher-level CQ individuals are not only more able to understand cultural differences but also to utilize them in more productive ways, leading to building relationships and even trust [49]. 

As referred, the present research focused on international SMEs’ top managers’ ENB and on their CQ as a significant antecedent. CQ emerges as a fundamental competence within the increasing cultural interactions in business reality, with its cultural differences, challenges, and complexities [47]. Taking the above into consideration, we assumed that CQ was a predictor of ENB and its dimensions. Higher-level CQ individuals perform better than others in terms of their ENB. CQ should help to capitalize on decision-makers’ actions toward ENB. Therefore, we formulated Hypothesis 2 (H2) as the following:

**Hypothesis 2** **(H2).** *The higher the level of a decision-maker’s CQ, the better their performance regarding their ENB.*

Earley and Mosakowski [47] stated that “cultural intelligence is related to emotional intelligence, but it picks up where emotional intelligence leaves off” (p. 139). Accordingly, high levels of EI within a specific culture are not necessarily converted into a successful adaptation to different cultural contexts [19]. Taking this into account, the emotional capability underlying EI is not automatically reflected in different cultural contexts [50]. In addition, and as it was stressed, CQ is a facilitator capability that allows for interacting well in intercultural settings [32], allowing managers to communicate better with others and promoting superior relationships [22]. CQ improves the effectiveness of intercultural negotiation processes and allows individuals to be more confident, motivated, and cooperative in intercultural relationships [32]. Lin et al. [21] showed the positive effect of CQ on cross-cultural adjustment. Therefore, due to the important value of CQ in international contexts, we formulated Hypothesis 3 (H3) as the following:

**Hypothesis 3** **(H3).** *In the international context, a decision-maker’s CQ is a more significant predictor of ENB than their EI.*

### 2.4. Relationship between the Dimensions of the Constructs and the Final Research Model Overview

Taking into consideration the multidimensional nature of the constructs, we intended to go deeper regarding our analysis concerning the relationship between the dimensions of EI and CQ with the dimensions of the top managers’ international ENB. We took the external networking behaviors to include the following three dimensions: building and maintaining external contacts (BMEC), using external contacts in a win–win perspective (UECWW), and commitment to creating external contacts (CCEC). The EI was composed of self-emotions appraisal (SEA), others-emotions appraisal (OEA), use of emotion (UOE), and regulation of emotion (ROE). Therefore, we suggest that those who better assess and learn about their own emotions (SEA), thereby promoting better social interactions [51], are better prepared to build and maintain external contacts, to use external contacts in a win–win perspective, and to commit to creating external contacts. Concerning others-emotions appraisal, we proposed that the better the top managers’ perception and understanding of others’ emotions [38] is, as well as their capability to create empathy and more fruitful relationships [51], the better they will build and maintain external contacts, use external contacts in a win–win perspective and commit to creating external contacts. The use of one’s emotional capability promotes reasoning, problem-solving, and interpersonal communication [39], as well as a greater encouragement to continuously do better [52]. The regulation of emotion allows individuals to regulate their emotions, thereby preventing, reducing, enhancing, or modifying self-emotional responses [39]. Thus, we proposed that these last two emotional capabilities allowed the international top managers to better build and maintain external contacts, to better use external contacts in a win–win perspective, and to better commit to creating external contacts. In relation to CQ, this intelligence integrated the metacognitive (MC), cognitive (COG), motivational (MOT), and behavioral (BEH) dimensions. Therefore, we suggested that the dimension that empowers top managers with the cultural awareness that allows them to draw up cognitive strategies and enhance their awareness about cultural interactions (MC) [48], as well as the dimension that reflects the knowledge in different cultures (COG) [50], making it possible to identify the different cultural characteristics within multicultural contexts [22], will positively affect the top managers, causing them to do better at building and maintaining their external contacts, using their external contacts in a win–win perspective, and better committing to creating external contacts. We finally proposed that these external networking behaviors may be improved due to the international decision-makers’ capabilities regarding their direct attention and energy that is dedicated to diverse cultural contexts (MOT) [48], as well as their ability to act appropriately and to adapt verbal and nonverbal behaviors in intercultural interactions (BEH) [50].

In this sense, we formulated hypotheses four (H4) to nine (H9) as the following:

**Hypothesis 4** **(H4).** *The higher the level of a decision-maker’s EI dimensions (SEA, OEA, UOE, and ROE), the better the performance of their ENB regarding building and maintaining external contacts (BMEC).*

**Hypothesis 5** **(H5).** *The higher the level of a decision-maker’s EI dimensions (SEA, OEA, UOE, and ROE), the better the performance of their ENB regarding using external contacts in a win–win perspective (UECWW).*

**Hypothesis 6** **(H6).** *The higher the level of a decision-maker’s EI dimensions (SEA, OEA, UOE, and ROE), the better the performance of their ENB commitment to creating external contacts (CCEC).*

**Hypothesis 7** **(H7).** *The higher the level of a decision-maker’s CQ dimensions (MC, COG, MOT, and BEH), the better the performance of their ENB regarding building and maintaining external contacts (BMEC).*

**Hypothesis 8** **(H8).** *The higher the level of a decision-maker’s CQ dimensions (MC, COG, MOT, and BEH), the better the performance of their ENB regarding using external contacts in a win–win perspective (UECWW).*

**Hypothesis 9** **(H9).** *The higher the level of a decision-maker’s CQ dimensions (MC, COG, MOT, and BEH), the better the performance of their ENB commitment to creating external contacts (CCEC).*

**Figure 1:**
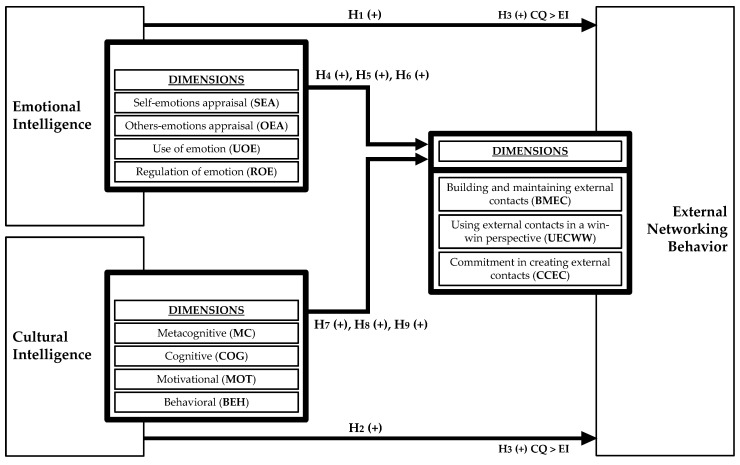
Research model overview.

According to the formulated hypotheses (H1 to H9), we present the following Figure 1 with the research model.

## 3. Empirical Study

### 3.1. Data and Sample

This research focused on decision-makers’ external networking behaviors in the international business field. Therefore, to study their external networking behaviors in international business environments, data was collected from the decision-makers directly responsible for international activities in Portuguese international SMEs. The sample was composed of founders, owners, chief executive officers (CEOs), managers of international activities, international market managers, or commercial managers. As a criterion for inclusion in the study, we requested the position of each respondent and followed the European Union’s definition of SMEs [53]. In the preliminary data examination, the respondents with incomplete information and the responses that did not comply with the SME criteria were excluded. Additionally, two respondents were eliminated from the database due to unengaged responses. Their answer variability was very close to zero [54] and a cutoff of less than 0.3 in the standard deviation of the responses was applied [55]. Therefore, 307 responses met the necessary criteria for further analysis. Regarding the missing data, we identified one case relating to the control variable “year of birth” of the respondent and one case in the respondents’ international experience (RIE). In these cases, we adopted the mean of the position group of the respondent. In relation to outliers, no questions were assumed in this subject, except in one observation regarding the RIE, where the value of the respondent seemed to be a typing error, which was replaced by the mean of the position occupied by the individual. In terms of the normality assumption, with samples of 200 cases or more, non-normality issues tend to be diminished [56]. The following Table 1 addresses the sample analysis in regard to the participants’ position, gender, and age.

The average age of participants was approximately 50 and 45 years old, for males and females, respectively, and the average of the respondents’ international experience was living in 1.2 countries.

### 3.2. Procedure

The survey, as a data collection instrument, was pretested by a panel of 10 individuals representing managers in internationalized enterprises. Minor changes were made in a few items. The data was collected from 2–30 April 2019, exclusively through online surveys sent by e-mail. In the survey development, we used previously validated scales with minor adaptations. All scales were in the English language but the sample was made up of native Portuguese speakers. To carry out this research in the Portuguese context, in relation to EI, we used the Portuguese translation of the Wong and Law Emotional Intelligence Scale (WLEIS) by Rodrigues et al. [57]. In the case of CQ, we used the Cultural Intelligence Scale (CQS) of Ang et al. [48], which was translated into Portuguese by Sousa et al. [58]. The ENB and Social Desirability (SD) scales were translated from English into Portuguese. We used the Behling and Law [59] translation/back-translation procedure with two bilingual scholars in Portuguese and English. The versions were compared, a new interaction was required, and final minor adjustments were made in a few items.

In business management research, the use of self-reporting measures is prone to bias, where the appearance of the social desirability effect in the responses is a reality that must be controlled [60]. Given the fact that this research used self-report measures, we used SD to control for the socially desirable response tendencies [61]. This study also controlled for age and gender. The respondents’ ages were controlled within the intelligence field, given that, for instance, EI increases with age [4]. Age and gender may influence networking behaviors [18]. Gender is also a widely used control variable within EI and CQ studies, given that it may influence them [31,62]. Concerning the potential influence of managers’ international experience on CQ [19], and as a source of knowledge to deal with the international environments [63], this research measured the respondents’ international experience (RIE). 

### 3.3. Portuguese SMEs

SMEs have high economic and social relevance and are considered the engine of the entire European economy [53]. Studying international firms, namely exporters, is a recognized field of research in countries like Portugal, where the export activity is decisive for the country’s economic growth [64]. This study focused on the Portuguese international SMEs’ business context. In the Portuguese context, exports have grown and played a major role in rebalancing the economy [65]. Indeed, for small economies, “internationalization is essential for country growth” [66] (p. 183). In this sense, Hortinha et al. [67] stated that “for Portuguese companies, exporting is a condition of survival, not only because of the current economic crisis but also because of the country’s small market” (p. 42). SMEs’ internationalization is a growing phenomenon [12] and is important for the development of this type of firm [68].

It should be noted that according to the observatory of economic complexity, in 2018, Portugal was the 44th largest export economy in the world [69]. Therefore, given the importance of SMEs’ internationalization to the Portugal business context, and the relevant role that networking has within the internationalization of firms, it has become highly pertinent to analyze the performance capacity of the international top managers in their external networking behaviors, as well as the psychological characteristics that can boost those behaviors. In the international context, emotional and cultural vicissitudes may emerge for those managers in charge of the firms’ international activities due to the highly multicultural environments that they have to deal with. Thus, the present study took emotional intelligence and cultural intelligence as the top managers’ major characteristics that influence their ability to perform their external networking behaviors. 

According to the European Commission [65], the Portuguese context has the potential to promote a greater internationalization of SMEs. Therefore, understanding the role of the international top managers’ characteristics in their international ENB is highly relevant to the firms’ international activities. Those behaviors may help managers to recognize and exploit new opportunities and promote more and better international sales. In summary, with the application of this study in the Portuguese international SMEs’ context, we intended to understand whether the psychological characteristics in this research helped to improve the international top managers’ international behaviors regarding building and maintaining external contacts, using external contacts in a win–win perspective, and in the commitment to creating external contacts.

### 3.4. Measurement

#### 3.4.1. External Networking Behavior

Networking is a complex construct with operationalization and measurement difficulties [3]. The present research followed a multidimensional approach. As referenced by Wolff and Moser [70], in the article “Entwicklung und Validierung einer Networkingskala (Development and validation of a networking scale),” which was published in the journal *Diagnostica* in 2006, Wolff and Moser developed a 44-item German measure of networking. This scale distinguishes between internal and external networking behaviors, with twenty-two items each [40]. In each one, the scale is a multidimensional one, composed of building contacts, maintaining contacts, and using contacts [40]. Concerning the original scale, ENB has seven items on building external contacts, seven items on maintaining external contacts, and eight items on using external contacts [28,40]. Given that it facilitates a more parsimonious survey, we used and adapted the ENB nine-item scale applied by Naudé et al. [24] to assess the networking of the international top managers with others outside the firm. Each dimension, namely, building external contacts (BEC), maintaining external contacts (MEC), and using external contacts (UEC), has three items. At this point, we should state that some modifications concerning the ENB factorial structure will emerge in the results section. In this respect, we found three dimensions that required an interpretation that was different from the original one. After the interpretation, we designated the first factor as building and maintaining external contacts (BMEC), the second factor as using external contacts in a win–win perspective (UECWW), and the third one as a commitment to creating external contacts (CCEC).

#### 3.4.2. Emotional Intelligence

In the present research, the scale of EI was a self-reported psychometric measure under the theoretical framework of EI as an ability [71], following Salovey and Mayer’s [51] and Mayer and Salovey’s [72] four-dimensional modeling definition. To measure EI, we used the scale of sixteen items developed by Wong and Law [36]. The WLEIS evaluates the overall construct of EI and its four dimensions: self-emotions appraisal (SEA), others-emotions appraisal (OEA), use of emotion (UOE), and regulation of emotion (ROE) [36,52]. This scale is widely used in the literature [24,37]. A high score on WLEIS reflects greater levels of EI [73].

#### 3.4.3. Cultural Intelligence

To measure CQ, we used Ang et al.’s [48] self-report psychometric four-dimensional scale with twenty items, known as the CQS, which follows Earley and Ang’s [5] definition of CQ. This scale evaluates CQ as a multidimensional construct composed of four dimensions: metacognitive (MC), cognitive (COG), motivational (MOT), and behavioral (BEH), which supports the overall construct of CQ [48]. This is a widely used scale for assessing CQ [22,23,31,32]. A high score on the CQS reflects the ability to better adjust, understand, and behave in new cultures [21].

These three constructs (ENB, EI, and CQ) were measured on a five-point Likert scale response format with 1—strongly disagree, 2—disagree, 3—neither agree nor disagree, 4—agree, and 5—strongly agree.

#### 3.4.4. Control Variables

*Social Desirability (SD)*

With the use of self-reported measures, it is crucial to measure the socially desirable response tendency, whether in the context of personality, affection, attitudes, and other individual characteristics [74]. To this end, Reynolds [74] developed three short versions of the Marlowe Crowne Social Desirability Scale (MCSDS). We used Reynolds’s [74] form C to assess the SD. Despite its thirteen items with a true/false response format [75], other studies used a Likert scale response format [76]. We used a five-point Likert scale response format like in the above constructs. The false-oriented items were subject to a reverse score.

*Age*

The age of the managers is seen as a proxy for their cognitive abilities and knowledge [77]. In the field of intelligences, age may reflect a positive correlation, e.g., with EI, as stated by Law et al. [52]. We used the year of birth to measure the age of the respondent.

*Gender (G)*

Gender may influence the intelligences in the present research; thus, we used it as a control variable, as in other studies in this area [31,38,48]. It was coded as 1—male and 2—female [38].

*Respondents’ International Experience (RIE)*

The international experience of managers influences their knowledge, and despite the existence of more accurate measures of international experience [63], we asked for the following information according to Rockstuhl et al. [78]: please indicate the number of countries where you have lived.

*Position*

To participate in this research, the respondents had to hold one of the following positions and indicate which one: founder, owner, CEO, manager of international activities, international market manager, or commercial manager.

### 3.5. Common Method Bias

This research took some steps to control and avoid the common method bias (CMB). It should be noted that the same individual provided all the collected data for each firm [79]. Following Podsakoff et al. [79], we took into consideration the respondent’s social desirability tendency and the potential existence of item complexity or ambiguity. We also informed the participants about the confidentiality of the responses, the requested honesty, and the non-existence of right or wrong answers [79]. In addition to the above procedures, and due to the fact that CMB may be a concern, we performed Harman’s single-factor test according to Podsakoff et al. [79]. With this test, 10 factors emerged with eigenvalues greater than one. No single factor emerged, and the first one did not account for the majority of the variance. Therefore, according to Podsakoff et al. [79], CMB was not a major problem in this research. Additionally, in order to control for CMB, the analysis of social desirability integrated the hypotheses testing as a control variable to mitigate its effects [80].

## 4. Results

The collected data were processed using the IBM SPSS Statistics of the Statistical Package for Social Science program version 25, Armonk, New York, United States of America.

### 4.1. Exploratory Factor Analysis

We performed an exploratory factor analysis on all the constructs in the study. All dependent, independent, and control variables were tested using the principal components extraction method with a varimax rotation.

Concerning the adapted ENB nine-item scale, according to the exploratory factor analysis, we retained three factors, taking into consideration that there was a total explained variance of 60.69%. That was the criterion that we used for factor extraction. In the field of the social sciences, a factor structure that accounts for an explained variance of 60% (or even less) is considered satisfactory [56]. After the varimax rotation, the three-factor structure obtained (Table 2) was different from the initial ENB scale. Hence, we found three dimensions that, despite not contradictory to the original scale, required a different interpretation of the original one that provided an interesting approach from the point of view of networking and international networking. Following the fundamental procedure of analyzing the content of the items, the final factor solution was interpreted as follows. The first factor was composed of item one of building external contacts (BEC), items two and three of maintaining external contacts (MEC), and item one of using external contacts (UEC). As Table 2 reflects, after the interpretation, we designated factor one as building and maintaining external contacts (BMEC). The second factor was composed of item one of MEC and items two and three of UEC. After the interpretation, we designated this factor as using external contacts in a win–win perspective (UECWW). The final factor was composed of items two and three of BEC. After the interpretation, we designated this factor as a commitment to creating external contacts (CCEC). Therefore, despite not being a radical and extremely different structure, it constituted an interesting approach with new insights for future research. These three factors were extracted, and their associated scores were saved for hypothesis testing.

To verify the adequacy of the collected information for factor analysis, we used the Kaiser–Meyer–Olkin (KMO) measure and Bartlett’s sphericity test. According to the observed values for all constructs (Table 3), pursuing a factor analysis was highly recommended [81]. As mentioned above regarding the ENB nine-item scale, we retained three factors (Table 3). From the sixteen EI items, the EI four-factor structure emerged with a total explained variance of 61.47%, verifying the foundations and theoretical principles of the WLEIS (see Table 3). The number of extracted factors was given by the Kaiser’s criterion and by a scree plot [81]. This scale measures EI and its four dimensions, specifically, SEA, OEA, UOE, and ROE [52]. These factors were extracted, and their associated scores were saved for hypothesis testing. We conducted an equivalent analysis regarding CQ. From the twenty CQ items, item number one in the MOT dimension exhibited cross-loadings. After trying different extraction methods with several rotations, we verified the persistence of this scenario and its elimination was performed [56]. After this elimination, we ran the analysis with the nineteen remaining items. An interpretable and significant factorial structure emerged according to the theoretical and empirical precepts of the CQS, with a total explained variance of 59.63%. This scale measured the four dimensions of CQ [48], specifically, MC, COG, MOT, and BEH. Their factor scores were saved for hypothesis testing. According to Hinton et al. [82], all the scales and factors exhibited moderate to high internal consistency (Table 3). Regarding the control variable SD with thirteen items, according to the KMO and Bartlett’s sphericity test, factor analysis was recommended. However, no unidimensional structure emerged. Given the true/false original response format, according to the original scale of Crowne and Marlowe [83], five items were true and eight items were false as part of a socially desirable response. Hence, two dimensions could be expected. Therefore, an exploratory factor analysis (EFA) was performed to define the extraction of two factors. After the factorial structure purification, the final rotated component matrix ended up with two factors and eight items. As a control variable, and due to the total SD scale (eight items) with a Cronbach’s alpha of 0.677 (Table 3), we computed a composite score of the average of the items. In summary, the total scale mean scores were calculated for ENB, EI, CQ, and SD.

### 4.2. Hypotheses Testing

We performed a correlation and descriptive analysis to get a first glimpse of the relationships between the variables. The results are reported in Table 4 for a sample size of 307 individuals.

The key variables of the research were significantly correlated (EI, CQ, and ENB). Age and gender did not reveal any significant correlations with EI, CQ, or ENB. Regarding SD, significant correlations were observable with EI and CQ. SD effects were controlled in the hypothesis testing. Finally, RIE exhibited significant correlations with CQ and SD.

Subsequently, to carry out the hypothesis testing, several hierarchical linear regression analyses with a confidence interval of 95% were performed, controlling for age, gender, RIE, and SD. Regarding the sample size adequacy, we observed the rule of five observations, as well as the preferable level of fifteen or even twenty observations for each independent variable [56].

*Hypothesis 1, Hypothesis 2, and Hypothesis 3*

According to the statistical F-test, the final regression models were significant at *p* < 0.001.

Hypothesis 1 stated that the higher the level of a decision-maker’s EI, the better their performance regarding their ENB. EI emerged as a significant positive predictor, accounting for 9.7% of the variation in ENB (see the Hypothesis 1 regression results in Table 5). We verified the expected positive relationship between EI and ENB. Therefore, Hypothesis 1 was supported. The international SMEs’ decision-makers’ EI emerged as a significant predictor of their ENB.

Hypothesis 2 stated that the higher the level of a decision-maker’s CQ, the better their performance regarding their ENB. CQ had a significant and positive effect, accounting for 13% of the variation in ENB (Table 5). We observed the expected positive relationship between CQ and ENB. Therefore, Hypothesis 2 was supported. It can be concluded that the international SMEs’ decision-makers’ CQ was a significant predictor of their ENB.

Hypothesis 3 stated that a decision-maker’s CQ is a more significant predictor of their ENB than the decision-maker’s EI. This hypothesis was supported by the results (Table 5): CQ (*β* = 0.280, *t*(300) = 2.452, *p* = 0.000) and EI (*β* = 0.200, *t*(300) = 2.809 *p* = 0.005).

In these regressions, regarding casewise diagnostics, none of the cases that deserved some attention exhibited a Cook’s distance greater than one or a centered leverage value greater than three times the calculated average value, and none were above an absolute value of one in terms of DFBeta statistics [81]. No influential cases were detected with an undue influence on the model [81]. Due to the verified correlations between the predictors, the variance inflation factor (VIF) values, and the T tolerance values, there were no multicollinearity concerns [81]. Concerning the VIF values, they are much lower than 10, indicating no cause for concern [81]. The independence of errors assumption was also observed in all regressions with the Durbin–Watson statistic (*d*) = 2.185, *d* = 2.156, and *d* = 2.163 for final model of H1, final model of H_2_, and final model of H_3_, respectively. The linearity and homoscedasticity assumptions and the normality of the residuals were confirmed [81].

*Hypothesis 4, Hypothesis 5, and Hypothesis 6*

The following regression results (Table 6) report the test results for hypotheses four, five, and six in terms of the four dimensions of EI and the three dimensions of ENB.

According to the results of Hypothesis 4, others-emotions appraisal (OEA), use of emotion (UOE), and regulation of emotion (ROE) significantly predicted the decision-makers’ engagement in building and maintaining external contacts (BMEC). A positive relationship was observable, as expected, between those EI dimensions and BMEC. Thus, as OEA, UOE, and ROE increased, so did BMEC. SEA was not statistically significant. Therefore, this hypothesis was partially supported, since SEA was not significant.

Hypothesis 5 stated that the higher the level of the EI dimensions of a decision-maker, the better their performance regarding ENB when using external contacts in a win–win perspective (UECWW). As expected, the use of emotion (UOE) exhibited a significant and positive effect on UECWW (Table 6). However, the other EI dimensions were not statistically significant. Therefore, Hypothesis 5 was partially supported.

Hypothesis 6 stated that the higher the level of the EI dimensions of a decision-maker, the better their performance regarding ENB in the commitment to creating external contacts (CCEC). As expected, the use of emotion (UOE) and the regulation of emotion (ROE) exhibited a significant and positive effect on CCEC (Table 6). SEA and OEA were not statistically significant. Therefore, Hypothesis 6 was partially supported.

Regarding casewise diagnostics, no influential cases were detected with an undue influence on the regressions. All the models’ assumptions were verified.

*Hypothesis 7, Hypothesis 8, and Hypothesis 9*

The following regression results (Table 7) report the test results of hypotheses seven, eight, and nine regarding the four dimensions of CQ and the three dimensions of ENB.

Hypothesis 7 stated that the higher the level of a decision-maker’s CQ dimensions, the better their performance regarding ENB when building and maintaining external contacts (BMEC). Metacognitive (MC), cognitive (COG), and motivational (MOT) dimensions of CQ, as expected, had a significant and positive effect on BMEC (Table 7). The behavioral (BEH) dimension was not statistically significant. Therefore, this hypothesis was partially supported.

Hypothesis 8 stated that the higher the level of a decision-maker’s CQ dimensions, the better their performance regarding ENB when using external contacts in a win–win perspective (UECWW). Motivational (MOT) and behavioral (BEH) dimensions had a significant and positive effect on using external contacts in a win–win perspective (Table 7). Therefore, this hypothesis was partially supported.

Hypothesis 9 stated that the higher the level of a decision-maker’s CQ dimensions, the better their performance regarding ENB in the commitment to creating external contacts (CCEC). The cognitive (COG) dimension was the main predictor, with a significant and positive effect on CCEC (Table 7). This hypothesis was partially supported.

Regarding casewise diagnostics, no influential cases were detected with an undue influence in the regressions. SD did not present any concerns across the models. All the models’ assumptions were verified.

In order to summarize the hypotheses testing, Table 8 provides an overview of the status of all the hypotheses.

## 5. Discussion

In this research, we tested international decision-makers’ psychological characteristics as the main predictors of their networking behaviors. We took cultural intelligence and emotional intelligence as significant characteristics that boost international decision-makers’ external networking behavior in the SMEs’ internationalization contexts. According to the results, this investigation showed the pivotal role of CQ and EI in external networking behavior. As expected, EI had a positive influence on ENB, indicating that the higher the capability of the decision-makers to reason about emotions and to use emotional knowledge to enhance thought [4], the better the performance of their behaviors when building and maintaining external contacts, when using external contacts in a win–win perspective, and in the commitment to creating external contacts. Cultural intelligence, as expected, also exhibited a positive effect on ENB, indicating the positive predictive value of international decision-makers’ capability to adapt to new cultural contexts. Therefore, the important role of EI and CQ in the international business management context of top managers’ external relationships should be stressed. Another important result concerned the fact that in the international business management context, the CQ capability was more significant to the top managers’ ENB than EI. Still, as already stated, this result did not invalidate the important role of EI in this context.

The present research also studied the value of the different EI and CQ dimensions on the new retained ENB dimensions. Hence, concerning EI, the results showed that others-emotions appraisal (OEA), use of emotion (UOE), and regulation of emotion (ROE) were the relevant variables in the context of the ENB of international top managers of international SMEs. Others-emotions appraisal is concerned with the capability for the perception and understanding of the emotions of others; the ability to predict the emotional responses of others [38]; the creation of empathy; the promotion of healthier, more satisfying, and fruitful relationships; the possibility of being better accepted [51]. According to the results, this emotional capability allowed international decision-makers to better build and maintain their external contacts. Using emotion to enhance cognitive activities is related to the ability to promote reasoning, problem-solving, and interpersonal communication [39]. This ability promotes the possibility of better individual encouragement to continuously do better [52]. Therefore, according to our results, using emotions adequately in the international business management context allowed international decision-makers to better establish relationships with others outside the organization in diverse aspects, namely, when building and maintaining their external contacts, when using their external contacts in a win-win perspective, and in their commitment to creating external contacts. The regulation of emotion also had a positive effect, showing that the better the top managers regulated their emotions via preventing, reducing, enhancing, or modifying self-emotional responses [39], the more capable they were to build and maintain external contacts and to commit themselves to creating contacts.

In relation to CQ, our results showed that its four dimensions were relevant in the context of the ENB of the SMEs’ international decision-makers. The metacognitive dimension empowers individuals with the cultural awareness that allows them to draw up cognitive strategies and enhance awareness about cultural interactions [48]. Therefore, this cultural consciousness allows decision-makers to better build and maintain external contacts in international contexts. Regarding the cognitive (COG) dimension, top managers with a high level in this capability are better prepared to identify the different cultural characteristics between different cultural contexts [22]. This dimension “reflects knowledge of norms, practices, and conventions in different cultures” [50] (p. 5). Consequently, individuals with higher levels in this dimension are better prepared to interact with different cultural settings [50], and following our results, due to this capability, they were more capable at building and maintaining external contacts and committing themselves to creating contacts. According to the results, individuals were better prepared to build and maintain contacts, as well as to use their external contacts in a win–win perspective due to their motivational capability. Individuals with higher motivational levels are more likely to direct attention and energy to diverse cultural contexts [48], to adapt themselves to different cultural contexts to overcome inherent difficulties [21], and are more prone to foster intercultural contacts and adapt behaviors [32]. As a final result, the behavioral (BEH) dimension, which is the capability to act appropriately and to adapt verbal and nonverbal behaviors in individual intercultural interactions [50], emerged as a significant predictor of the decision-makers when using their external contacts in a win–win perspective.

### Implications

This study has several theoretical and practical implications for our understanding of the role that cultural intelligence and emotional intelligence plays in improving the engagement of the top managers’ external networking behavior within the international business management context.

#### 5.1.1. Theoretical Implications

Overall, this research makes three major theoretical contributions. First, given the importance of networking within the international business management context, our study’s findings contribute to network theories suggesting that the top managers’ psychological characteristics have a central role in their external networking behavior. Second, the present study also contributes to emotional intelligence theory and to cultural intelligence theory by showing the booster value of these intelligences to the international top managers’ external networking behavior in international business contexts. Therefore, the present research makes these contributions at the confluence of the international business, networks, and psychological fields. Lastly, and adding to these theoretical bodies of knowledge, interesting theoretical implications emerged concerning the relationship between the emotional intelligence and cultural intelligence dimensions and the new retained external networking behavior’s factorial structure, as stated above.

As a relatively new approach, this research adds to the literature on emotional intelligence theory, cultural intelligence theory, and network theory within the international business management body of knowledge, suggesting that the higher the levels of international top managers’ emotional and cultural intelligences, the better the performance of their external networking behavior.

#### 5.1.2. Practical Implications

The results of our research are of interest to the international management field. The findings shed light on the value of the emotional and cultural intelligences of the firms’ international decision-makers. According to our results, emotional intelligence and cultural intelligence play an important role in the top managers’ external networking behavior. In line with this, we offered insights into the psychological characteristics that may play an important role in the performance of the top managers’ behaviors when building and maintaining external contacts, when using external contacts in a win–win perspective, and in the commitment to creating external contacts. These findings have important implications for international top managers’ profiles. The results showed that the higher the capability of the decision-makers to reason about emotions, to use emotional knowledge to enhance thought, and to adapt to new cultural settings, the better their chance of engaging better in their external networking behavior. In this sense, international firms that want to have capable international top managers to engage and improve their external networking behaviors should provide training programs on emotional and cultural intelligences to their major international human resources. Specific training programs should be provided to improve their different emotional and cultural capabilities.

From a dimensional perspective, our findings also indicated that in order to better build and maintain their external contacts, international top managers must be aware and improve their emotional capabilities concerning the perception and understanding of the emotions of others, the use of emotions to facilitate performance, and the regulation of emotions. To this end, they could also improve their cultural capabilities concerning their cultural awareness, their cultural knowledge, and their capability to direct attention and energy to diverse cultural contexts. Our results suggest that the international SMEs’ top managers had a greater chance of using their external contacts in a win–win perspective if they invested themselves in improving their use of their emotional capability and their cultural motivational and behavioral capabilities. Concerning the commitment to creating external contacts, we encourage international managers to improve their cultural knowledge and their emotional capabilities for using and regulating emotions. Therefore, an important managerial implication concerns the fact that the two intelligences in question are trainable and might be improved by the international top managers to produce better external networking behavior. Given the importance of networking in the international business context, the organizations that are internationally active or those that are intending to approach the international markets should assess the cultural and emotional intelligences of their potential international top managers, not only when hiring new ones but also when promoting internally. These are also important intelligences for teamwork development.

Thus, this research shed light on the role of the emotional and cultural intelligences of the founders, owners, chief executive officers (CEOs), managers of international activities, international market managers, or commercial managers of the international SMEs in the particular case of Portugal. Improving these intelligence levels would allow international decision-makers to enhance their external networking behavior.

## 6. Conclusions

The importance of the relationships through networks is recognized within the SMEs’ international context [15]. In this context, we emphasized the important role of top managers’ psychological characteristics. In fact, networking activity is influenced by individual differences [18], where the importance of the individuals’ psychological characteristics as antecedents has been suggested [3]. In this vein, we showed the pivotal role of CQ and EI in international decision-makers’ ENB. They are two important intelligences within cross-cultural contexts [19]. Therefore, we provided important evidence within networking, cultural intelligence, and emotional intelligence fields, demonstrating the predictive value of these two intelligences.

In relation to the exploratory analysis of ENB, we found three different factors that embodied an interesting approach. Hence, after the interpretation, we retained the following three dimensions: building and maintaining external contacts, using external contacts in a win–win perspective, and a commitment to creating external contacts. To deepen the analysis, we also studied the value of the decision-makers’ cultural and emotional intelligence dimensions on the external networking behavior factors used when conducting the empirical study. Important relations between CQ and EI dimensions and the new retained factor structure of ENB constituted an important result of our study.

In summary, this research suggested that cultural and emotional capabilities help top managers to engage in multicultural contexts in order to achieve better results in their ENB.

Regarding limitations, we emphasize the main ones, starting by mentioning that the self-report nature of the measures may lead to biases. However, its use is widely seen in the study of intelligences and external networking behavior [21,24,28,32,48]. Concerning this issue, for further research, we emphasize the need, as reflected by many scholars, to use tests to assess ability levels [52]. It is worth noting that our study was based on cross-sectional data, a fact that disturbs the assessment of causality between constructs [22]. Therefore, longitudinal studies promote another type of assessment of the relationships between the variables. To improve the CQ body of knowledge, one suggestion to further research is the application of the CQ expanded scale of Van Dyne et al. [84]. Along this line, another suggestion for further research is to consider the business cultural intelligence quotient model of CQ developed by Alon et al. [46] to undertake investigations related to business and international business management contexts. Following Leonidou et al. [29], due to the fact that relationships are a dynamic phenomenon, longitudinal studies appear to allow for the understanding of their evolution and the role of EI and CQ in the different stages of the relationships. Given this dynamic nature, quantitative and qualitative studies should complement each other in a richer approach, promoting a better understanding of the networking phenomenon [45]. In order to improve the EI, CQ, and ENB bodies of knowledge, we suggest the application of this study in different cultural realities, in other countries, and in larger companies. As a final limitation, we emphasize that this study was carried out in the specific context of the Portuguese economy, a fact that may not allow its generalization to other economic environments.

## Figures and Tables

**Table 1:** Sample analysis (participants’ position, gender, and age).

Position/Gender	Proportion	Age (Average)
Founders and owners	46.9%	51
Male	35.5%	51
Female	11.4%	50
CEOs	21.8%	51
Male	16.9%	52
Female	4.9%	45
Commercial managers	13.7%	43
Male	7.2%	44
Female	6.5%	41
Managers of international activities	13.0%	43
Male	8.5%	45
Female	4.6%	38
International market managers	4.6%	40
Male	3.3%	40
Female	1.3%	39
Total	100%	48

CEOs: chief executive officers.

**Table 2:** Exploratory factor analysis matrix for external networking behavior (ENB).

Scale Items and Factorial Structure	BMEC	UECWW	CCEC
**BEC 1:** I develop informal contacts with professionals outside the organization, both nationals and foreigners, in order to have personal links beyond the company.	0.756		
**BEC 2:** I take part in national and international professional meetings.			0.830
**BEC 3:** I use business trips and other national and international programs to build new contacts.			0.781
**MEC 1:** When I obtain informal information that might be of importance to partners from other organizations, I pass it on to them.		0.682	
**MEC 2:** I use my contacts outside my company to ask for business advice.	0.755		
**MEC 3:** For business purposes, I keep in contact with former national and international partners.	0.585		
**UEC 1:** If I meet partners from other national and international organizations, I approach them to catch up on news and changes in their professional lives.	0.593		
**UEC 2:** I exchange professional tips and hints with partners from other national and international organizations.		0.705	
**UEC 3:** When I cannot solve a problem at my company, I call partners from other organizations and ask for advice.		0.787	

BMEC: building and maintaining external contacts, CCEC: commitment to creating external contacts, UECWW: using external contacts in a win–win perspective.

**Table 3:** Exploratory factor analysis report (EI, CQ, ENB, and SD).

Heading	K Items	Kaiser–Meyer–Olkin (KMO)	Bartlett’s Sphericity Test	Cronbach’s Alpha (α)
**Emotional intelligence (EI) (16 items)**	**16**	**0.848**	**0.000**	**0.865**
SEA: Self-emotions appraisal	4	-	-	0.757
OEA: Others-emotions appraisal	4	-	-	0.721
UOE: Use of emotion	4	-	-	0.700
ROE: Regulation of emotion	4	-	-	0.874
**Cultural intelligence (CQ) (19 items)**	**19**	**0.887**	**0.000**	**0.888**
MC: Metacognitive	4	-	-	0.792
COG: Cognitive	6	-	-	0.852
MOT: Motivational	4	-	-	0.760
BEH: Behavioral	5	-	-	0.792
**External networking behavior (9 items)**	**9**	**0.850**	**0.000**	**0.783**
BMEC: Building and maintaining external contacts	4	-	-	0.713
UECWW: Using external contacts in a win–win perspective	3	-	-	0.652
CCEC: Commitment to creating external contacts	2	-	-	0.553
**Social desirability (SD) (8 items)**	**8**	**0.779**	**0.000**	**0.677**
SDF: False-oriented items	5	-	-	0.782
SDT: True-oriented items	3	-	-	0.569

**Table 4:** Descriptive statistics and correlations.

Variables	Mean	Standard Deviation	EI	CQ	ENB	SD	G	Age	RIE
EI	3.89	0.42	1	0.526 ^**^	0.285 ^**^	0.496 ^**^	0.017	0.033	0.055
CQ	3.71	0.42	0.526 ^**^	1	0.359 ^**^	0.191 ^**^	0.017	−0.033	0.163 **
ENB	3.54	0.57	0.285 ^**^	0.359 ^**^	1	0.026	−0.013	−0.029	0.021
SD	3.59	0.55	0.496 ^**^	0.191 ^**^	0.026	1	0.044	0.188 ^**^	0.140 *
G	1.29	0.45	0.017	0.017	−0.013	0.044	1	−0.211 ^**^	−0.053
Age	48.15	10.85	0.033	−0.033	−0.029	0.188 ^**^	−0.211 ^**^	1	0.091
RIE	1.22	1.92	0.055	0.163 **	0.021	0.140 *	0.053	0.091	1

**: Correlation was significant at the 0.01 level (two-tailed). *: Correlation was significant at the 0.05 level (two-tailed). G: gender, RIE: respondents’ international experience.

**Table 5:** Regression results (H1, H2, and H3).

Variables Entered	Hypothesis 1			Hypothesis 2			Hypothesis 3		
	Final Model			Final Model			Final Model		
	ENB			ENB			ENB		
	*β*	*t*	Sig.	*β*	*t*	Sig.	*β*	*t*	Sig.
**Block 1: Control variables**									
Age	−0.017	−0.295	0.768	−0.011	−0.190	0.849	−0.005	−0.087	0.931
Gender (G)	−0.014	−0.257	0.797	−0.021	−0.390	0.697	−0.018	−0.321	0.748
Respondents’ international experience (RIE)	0.023	0.416	0.678	−0.034	−0.627	0.531	−0.019	−0.350	0.727
Social desirability (SD)	−0.152	−2.347	0.020	−0.038	−0.666	0.506	−0.123	−1.936	0.054
**Block 2: Key variables**									
Emotional intelligence (EI)	0.360	5.708	0.000	-	-	-	0.200	2.809	0.005
Cultural intelligence (CQ)	-	-	-	0.372	6.706	0.000	0.280	4.396	0.000
*R* ^2^		0.100			0.132			0.155	
Adjusted *R*^2^		0.085			0.118			0.138	
∆*R*^2^		0.097			0.130			0.152	
F		6.694			9.178			9.138	
Sig.		0.000			0.000			0.000	
∆F		32.579			44.966			26.942	
Sig.		0.000			0.000			0.000	

All *β* are standardized coefficients.

**Table 6:** Regression results (H_4_, H_5_, and H_6_).

Variables Entered	Hypothesis 4			Hypothesis 5			Hypothesis 6		
	Final Model			Final Model			Final Model		
	BMEC			UECWW			CCEC		
	*β*	*t*	Sig.	*β*	*t*	Sig.	*β*	*t*	Sig.
**Block 1: Control variables**									
Age	0.072	1.253	0.211	0.042	0.711	0.478	0.005	0.092	0.927
Gender (G)	0.199	3.503	0.001	0.147	2.513	0.012	0.083	1.451	0.148
Respondents’ international experience (RIE)	0.020	0.366	0.714	−0.064	1.126	0.261	0.061	1.084	0.279
Social desirability (SD)	0.158	2.366	0.019	0.107	1.556	0.121	0.004	0.057	0.955
**Block 2: Key variables (EI dimensions)**									
Self-emotions appraisal (SEA)	0.085	1.506	0.133	0.060	1.045	0.297	0.004	0.077	0.939
Others-emotions appraisal (OEA)	0.110	1.974	0.049	0.101	1.766	0.078	0.097	1.736	0.084
Use of emotion (UOE)	0.164	2.781	0.006	0.152	2.513	0.012	0.204	3.434	0.001
Regulation of emotion (ROE)	0.199	3.297	0.001	0.010	−0.162	0.872	0.165	2.708	0.007
*R* ^2^		0.108			0.060			0.091	
Adjusted *R*^2^		0.084			0.035			0.067	
∆*R*^2^		0.061			0.032			0.059	
F		4.522			2.386			3.750	
Sig.		0.000			0.017			0.000	
∆F		5.132			2.562			4.844	
Sig.		0.001			0.039			0.001	

All *β* are standardized coefficients.

**Table 7:** Regression results (H7, H8, and H9).

Variables Entered	Hypothesis 7			Hypothesis 8			Hypothesis 9		
	Final Model			Final Model			Final Model		
	BMEC			UECWW			CCEC		
	*β*	*t*	Sig.	*β*	*t*	Sig.	*β*	*t*	Sig.
**Block 1: Control variables**									
Age	0.079	1.372	0.171	0.043	0.728	0.467	−0.008	−0.128	0.898
Gender (G)	0.212	3.742	0.000	0.156	2.714	0.007	0.059	1.027	0.305
Respondents’ international experience (RIE)	0.021	0.371	0.711	0.094	1.641	0.102	0.034	0.595	0.552
Social desirability (SD)	0.048	0.833	0.405	0.063	1.064	0.288	0.115	1.956	0.051
**Block 2: Key variables (CQ dimensions)**									
Metacognitive (MC)	0.124	2.207	0.028	0.003	0.047	0.962	0.039	0.694	0.488
Cognitive (COG)	0.153	2.754	0.006	0.110	1.952	0.052	0.174	3.091	0.002
Motivational (MOT)	0.158	2.835	0.005	0.146	2.567	0.011	0.097	1.709	0.089
Behavioral (BEH)	0.055	1.001	0.318	0.120	2.134	0.034	0.101	1.808	0.072
*R* ^2^		0.109			0.074			0.081	
Adjusted *R*^2^		0.085			0.049			0.057	
∆*R*^2^		0.062			0.046			0.049	
F		4.544			2.958			3.297	
Sig.		0.000			0.003			0.001	
∆F		5.174			3.675			3.967	
Sig.		0.000			0.006			0.004	

All *β* are standardized coefficients.

**Table 8:** Hypotheses final report.

Hypotheses	Findings	Status
H1	EI was a significant predictor of ENB	Supported
H2	CQ was a significant predictor of ENB	Supported
H3	CQ was a more significant predictor of ENB than EI	Supported
H4	OEA, UOE, and ROE positively predicted BMEC	Partially supported
H5	UOE positively predicted UECWW	Partially supported
H6	UOE and ROE positively predicted CCEC	Partially supported
H7	MC, COG, and MOT positively predicted BMEC	Partially supported
H8	MOT and BEH positively predicted UECWW	Partially supported
H9	COG positively predicted CCEC	Partially supported
